# Comparing Charlson and Elixhauser comorbidity indices with different weightings to predict in-hospital mortality: an analysis of national inpatient data

**DOI:** 10.1186/s12913-020-05999-5

**Published:** 2021-01-06

**Authors:** Narayan Sharma, René Schwendimann, Olga Endrich, Dietmar Ausserhofer, Michael Simon

**Affiliations:** 1 Institute of Nursing Science (INS), Department Public Health (DPH), Faculty of Medicine, University of Basel, Basel, Switzerland; 2 Patient Safety Office, University Hospital Basel, Basel, Switzerland; 3 Directorate of Medicine, Inselspital University Hospital Bern, Bern, Switzerland; 4 College of Health-Care Professions Claudiana, Bozen, Italy; 5 Nursing Research Unit, Inselspital University Hospital Bern, Bern, Switzerland

**Keywords:** Comorbidity indices, Weights, Risk adjustment, Inpatient data, In-hospital mortality

## Abstract

**Background:**

Understanding how comorbidity measures contribute to patient mortality is essential both to describe patient health status and to adjust for risks and potential confounding. The Charlson and Elixhauser comorbidity indices are well-established for risk adjustment and mortality prediction. Still, a different set of comorbidity weights might improve the prediction of in-hospital mortality. The present study, therefore, aimed to derive a set of new Swiss Elixhauser comorbidity weightings, to validate and compare them against those of the Charlson and Elixhauser-based van Walraven weights in an adult in-patient population-based cohort of general hospitals.

**Methods:**

Retrospective analysis was conducted with routine data of 102 Swiss general hospitals (2012–2017) for 6.09 million inpatient cases. To derive the Swiss weightings for the Elixhauser comorbidity index, we randomly halved the inpatient data and validated the results of part 1 alongside the established weighting systems in part 2, to predict in-hospital mortality. Charlson and van Walraven weights were applied to Charlson and Elixhauser comorbidity indices. Derivation and validation of weightings were conducted with generalized additive models adjusted for age, gender and hospital types.

**Results:**

Overall, the Elixhauser indices, c-statistic with Swiss weights (0.867, 95% CI, 0.865–0.868) and van Walraven’s weights (0.863, 95% CI, 0.862–0.864) had substantial advantage over Charlson’s weights (0.850, 95% CI, 0.849–0.851) and in the derivation and validation groups. The net reclassification improvement of new Swiss weights improved the predictive performance by 1.6% on the Elixhauser-van Walraven and 4.9% on the Charlson weights.

**Conclusions:**

All weightings confirmed previous results with the national dataset. The new Swiss weightings model improved slightly the prediction of in-hospital mortality in Swiss hospitals. The newly derive weights support patient population-based analysis of in-hospital mortality and seek country or specific cohort-based weightings.

**Supplementary Information:**

The online version contains supplementary material available at 10.1186/s12913-020-05999-5.

## Background

Critical health outcomes such as mortality often require effective risk adjustment based on patient characteristics to predict in-hospital mortality. This is also true for comorbidities [1, 2], which function as major predictors of mortality [3]. Over one-third of hospitalized patients have at least one comorbidity; two-thirds of those over 65 [2, 4] and three-quarters of those over 85 have at least two [5]. In addition to mortality, comorbidities are associated with lower health-related quality of life, increased disability and higher utilization of both health care services and prescribed medications [6–8].

Data on comorbidities are valuable both for comparison between patient populations and for risk adjustment regarding associated outcomes, especially mortality [9]. Two of the best-known measures are the Charlson Comorbidity Index and the Elixhauser Comorbidity Index [10, 11]. When the Charlson Comorbidity Index was developed in 1987 it included 19 chronic conditions to predict one-year mortality, but has since been shortened to 17. The Elixhauser Comorbidity Index, which was developed in 1998, works on a similar system but includes 30 – or, for some variants, 31 – comorbidities. In addition to in-hospital mortality, it is also used to predict the length of stay, adverse events and hospital discharges [12, 13]. Despite this additional versatility (covering acute and chronic conditions) and strong evidence that the Elixhauser Comorbidity Index is statistically superior to the Charlson Comorbidity Index [13, 14], the Charlson Comorbidity Index continues to be used. Because of the fewer chronic conditions [15–17] and comparative ease of use in routine situations where time is limited.

Both indices work either via simple (unweighted) sum scores or as weighted scores assigning a risk weight to each comorbidity [6, 18, 19]. A weighted sum score/summary measure provides an attractive advantage over plain dummy variables [20, 21], as it reduces the overfitting risk of more parameters, unjustifiable in small datasets [22] and limits computational requirements in large ones [21]. Additionally, evidence indicates that a weighted variable reduces type I errors compared to dummy variables while addressing multicollinearity concerns in regression analysis and organizing multiple highly correlated variables into more meaningful information [21, 23]. The weight assigned to each comorbidity reflects a higher, lower or neutral risk of mortality [24]. Practically, mortality risk scores can help to identify high-risk cases for special management and to assess provider services whose patients perform better or worse than expected from the summary measure of the morbidity burden.

To add to the value of early versions of the Elixhauser comorbidities, van Walraven et al. [25] used roughly 13 years’ inpatient admission data from one Canadian hospital (1996–2008) to develop a set of weights (VW weights, i.e., the regression coefficient divided by the coefficient in the model with the smallest absolute value) for the 30 Elixhauser comorbidities associated with in-hospital mortality. Using the backward selection and an alpha inclusion criterion of 0.05 to identify independently associated comorbidities, van Walraven identified 21 comorbidities significantly associated with mortality. A VW weight was assigned to each of the 21 Elixhauser comorbidities. Ultimately, VW weights ranged from − 7 to 12, with a weight of 0 assigned to the 9 non-significant comorbidities.

Since then, primarily in North America, studies have used VW weights to predict in-hospital mortality, especially in clearly defined patient groups such as surgical, orthopaedic, or cancer patients and those in single hospitals or intensive care unit (ICU) [12, 13, 21, 25, 26]. Moreover, the comorbidity weighting system might differ between all hospitalization and a restricted cohort; mortality and other outcomes; and between the countries [27]. Few studies have applied comorbidity adjustments to national or regional inpatient datasets [21, 28]. Therefore, an analysis of a large heterogeneous patient population from a national dataset (Switzerland) is justified both to provide an overview of Elixhauser comorbidities in a European sample and potentially to optimize the comorbidity weights. In addition to increasing the generalizability of these comorbidity weights, the use of such a dataset, representing all hospital inpatient cases (i.e., hospitalisation episodes) from a large, heterogeneous patient population, would allow a very accurate comparison of weighting systems. Therefore, the aims of our study were 1) to derive a new Swiss comorbidity weighting on a national inpatient dataset to predict in-hospital mortality; 2) to validate Charlson, Elixhauser-van Walraven and new weights on a national inpatient dataset; and 3) to compare the predictive performance of in-hospital mortality of the three weighting systems.

## Methods

### Study design and population

This is a retrospective population-based analysis of 6 years’ data (2012–2017) from the Swiss national inpatient dataset. Upon our application, subject to a data protection contract (as stipulated by article 22 of the Swiss Federal Act on Data Protection), the Swiss Federal Statistics Office (FSO) provided anonymized data from all Swiss hospital inpatient cases hospitalized between 2012 and 2017. This included not only general hospitals but also special care (e.g., paediatric, gynaecological) facilities [29]. The FSO classifies general hospitals (University hospitals, Tertiary hospitals, and three Basic hospitals) into five different levels, based on the number of cases treated per year and/or a special hospital score assigned by Swiss Medical Association (“FMH-Kategorien”). For this study, special care hospitals and children were excluded because of the low levels of comorbidities and the relatively low risk of dying in the hospital [25]. For data protection reasons, age was grouped in five-year groups, and all inpatient cases below 20 years of age were excluded. The flowchart for the final adult population included 102 general hospitals (6,094,672 inpatient cases) for the analysis is reported in supplementary Fig. F1 (Additional file 1).

### Dataset and classification of comorbidities indices

The dataset included patient characteristics including sex, age, hospital types, primary and secondary diagnoses based on International Classification of Diseases-10 (ICD-10) codes and hospital discharge information including in-hospital mortality. As condition coding in Switzerland is based on the ICD-10 German Modification (ICD-10 GM), reported in supplementary Table S1 (Additional file 1), we used this to identify both Charlson and Elixhauser comorbidities. Specifically, we used Quan et al.’s ICD-10 codes [19] to determine each of the 17 Charlson and 31 Elixhauser comorbidities via the “Comorbidity” package in R [18]. This transforms ICD-10 codes into binary data the relevant comorbidities, their (unweighted) sum scores, and their Charlson and VW-weighted scores.

### Descriptive analysis

The study population’s general characteristics (hospital types, patient’s sex, and age groups) were reported in the alive and mortality cohorts with percentages. The distributions of Charlson and Elixhauser comorbidities, unweighted and weighted scores were computed as percentages of index values of 0, 1–2, and ≥ 3 and < 0, 0, 1–4, and ≥ 5; and as the Charlson weight do not use negative weightings, its weights were calculated for index values of 0, 1–4, and ≥ 5. For each characteristic and comorbidities, standardized mean differences (SMD) between the alive and mortality cohort were computed using “tableone” package in R. The SMD is identical to Cohen’s D and provides an effect size estimate less sensitive to the sample size than *p* values between the cohorts. This is important in a dataset of the size used in this study. An SMD of zero means there is no difference in the characteristics (e.g., gender) between the alive and mortality cohort. SMDs greater than 0.1 indicate potentially relevant differences [30], i.e., showing unbalanced covariates and might have an association with mortality.

### Derivation of Swiss comorbidity weights

The study population was randomly split into a derivation (50%) and a validation (50%) group. The derivation group was used to determine the adjusted association of all 31 Elixhauser comorbidities with death, treating the anonymous hospital identifier as a random effect [31]. Generalized additive regression models (GAM) can accommodate many predictors including random effect, able to handle large dataset easily and nonparametric in nature [32]. We fitted GAM to compute the odds ratios (OR) using the package “mgcv” [33] and R programming language, version 3.5.2 [34]. We utilized GAM with random effect components on the hospital level, as university and small hospitals are different in size and services in Switzerland. To identify Elixhauser comorbidities associated with in-hospital mortality, we retained variables based on an alpha inclusion criterion of 0.01.

To derive the Swiss weightings from the regression model’s parameter estimates, we used the method described by Sullivan et al. [35]. Comorbidities not significantly associated with mortality were assigned a weight of zero. The number of (weighted) points assigned to each comorbidity equalled its regression coefficient divided by the coefficient in the model with the smallest absolute value [14, 21, 25, 35] rounded to the nearest whole number. Each person’s new Elixhauser comorbidity weighting score was then calculated by summing up all points of all their coded comorbidities.

### Validation and comparison of weighted comorbidity models

To validate and compare the performance of the three comorbidity weighting systems, we first created four multivariate in-hospital mortality prediction GAMs for the derivation group. The first model, ‘base’, contained no comorbidity data – only age group, sex, and hospital types. The other three models used the same variables as the base model, with the first, ‘Charlson’, using Charlson weights, the second, ‘van Walraven’, using the Elixhauser index with van Walraven weights, and the third, ‘Swiss weights’, using our newly-developed weights. We then validated all weights in validation groups by splitting the validation group into six groups by year of discharge. Altogether, 24 c statistics (including base models) were computed to validate the Charlson, van Walraven, and Swiss weights models in the validation sample. An additional four models were created using all cases (combining derivation and validation groups) to evaluate the performance of each model in the total patient population.

We assessed the various comorbidity weightings according to the model performance criteria. Discrimination, i.e., each model’s ability to distinguish patients discharged alive from those who died in hospital, was compared using the concordance (c) statistic. The c-statistic quantified each model’s ability to assign high probabilities of mortality to patients who died [36]. It’s possible values range from 0.50 to 1.0, with 0.50 indicating no ability to discriminate, values less than 0.70 are considered poor, those between 0.70 and 0.80 acceptable, and those of 0.80 or above excellent [37]. Using bootstrap methods, we computed 95% confidence intervals for each c-statistic. Additionally, the observed value was also explored for each model from the predicted values to observe the model performances in the highest selected percentages (1, 2, 5 and 10%) in the derivation sample. We also graphed receiver-operating characteristic curves (ROC) for the visual presentation of the derivation group’s c statistics. We compared the base model and existing comorbidity models with Swiss weight model using net reclassification improvement (NRI) for binary outcome [38–40] from the “nricens” package in R [41] using the Swiss derivation sample and classification cut-off value of 0.023 (mortality proportion of the total study population). NRI measures the degree of improvement in predicted inpatient mortality probabilities when comorbidity weights are added to the base model [21, 42]. Higher NRI values indicate more accurate reclassification.

### Code validation and sensitivity analyses

We also evaluated the R comorbidity package’s code handling accuracy in the Swiss setting. To do so we sampled 100 cases and manually reviewed the Swiss ICD-10 codes of the raw data, checking whether the “comorbidity” package had assigned each to the appropriate Charlson or Elixhauser comorbidity. We also performed sensitivity analyses to explore Switzerland’s Major Diagnostic Categories’ (MDCs’) associations, which are based on ICD-10 GM (one way of expressing the reason for admission), if any, regarding the change in the predictability of in-hospital mortality in combination with the above models and to test whether the combined models’ patterns differed from those of uncombined ones. MDCs are 24 mutually exclusive categories into which all primary diagnoses are assigned based on the Swiss diagnostic-related group (DRG) system for hospital reimbursement [43].

## Results

### Population characteristics

Overall, the adult inpatient population between 2012 and 2017 in all Swiss general hospitals (102) consisted of 6,094,672 cases. Among all hospitalized cases in our study population mortality was 2.3%. The characteristics of the adult inpatient cases are presented in Table 1. Inpatient cases had between 0 and 9 Charlson comorbidities (median 0, interquartile range (IQR): 0–1) and between 0 and 16 Elixhauser comorbidities (median 1, IQR: 0–2). The different categories of three comorbidity weightings are presented in supplementary Table S2 (Additional file 1).

**Table 1:** General characteristics of the total study population

Parameters	Alive cohort (%)	Mortality cohort (%)	SMD
^a^Total population: *N* = 6,094,672	5,952,005 (97.7)	142,667 (2.3)	
Females	3,280,823 (55.1)	63,912 (44.8)	0.208
Age groups			1.006
20–24 years	215,672 (3.6)	292 (0.2)	
25–29 years	327,562 (5.5)	375 (0.3)	
30–34 years	415,022 (7.0)	526 (0.4)	
35–39 years	348,591 (5.9)	718 (0.5)	
40–44 years	299,985 (5.0)	1368 (1.0)	
45–49 years	350,899 (5.9)	2503 (1.8)	
50–54 years	408,028 (6.9)	4312 (3.0)	
55–59 years	430,721 (7.2)	6503 (4.6)	
60–64 years	466,543 (7.8)	9068 (6.4)	
65–69 years	528,374 (8.9)	13,322 (9.3)	
70–74 years	554,612 (9.3)	16,899 (11.8)	
75–79 years	535,543 (9.0)	19,888 (13.9)	
80–84 years	509,225 (8.6)	24,853 (17.4)	
85–89 years	365,924 (6.1)	24,042 (16.9)	
90–94 years	161,236 (2.7)	14,156 (9.9)	
95+ years	34,068 (0.6)	3842 (2.7)	
Hospital types			0.157
University (level 1)	1,078,612 (18.1)	29,379 (20.6)	
Tertiary care (level 2)	3,274,382 (55.0)	83,686 (58.7)	
Basic care (level 3)	736,465 (12.4)	14,863 (10.4)	
Basic care (level 4)	671,182 (11.3)	10,695 (7.5)	
Basic care (level 5)	191,364 (3.2)	4044 (2.8)	
Number of Charlson comorbidities			1.234
0	3,642,650 (61.2)	17,465 (12.2)	
1–2	1,907,761 (32.1)	80,876 (56.7)	
> = 3	401,594 (6.7)	44,326 (31.1)	
Number of Elixhauser comorbidities			1.039
0	2,509,169 (42.2)	11,036 (7.7)	
1–2	2,106,780 (35.4)	43,494 (30.5)	
> = 3	1,336,056 (22.4)	88,137 (61.8)	

*Abbreviations: SMD* standardized mean difference between alive and mortality cohort

^a^Total population presented in row percentage

### Prevalence of Charlson and Elixhauser comorbidity indices

The most common Charlson comorbidity was any malignancy (including lymphoma and leukaemia, except malignant neoplasm of the skin) in both cohorts, alive (10.2%) and mortality (37.6%), yet with marked differences between the two cohorts (SMD: 0.680). The prevalence for each Charlson comorbidity in the total population and the derivation is presented in supplementary Table S3 (Additional file 1).

The most common Elixhauser comorbidities were uncomplicated hypertension (22.7%) in the alive cohort, whereas in the mortality cohort, it was solid tumour without metastasis (33.7%). However, the most pronounced difference between both cohorts was observed for metastatic cancer (4.0% vs. 26.5%; SMD: 0.657). The prevalence for each Elixhauser comorbidity from the total population and derivation group is presented in the supplementary Table S4 (Additional file 1).

### Derivation of Swiss weights

In the derivation group, two of the 31 Elixhauser comorbidities showed no association with hospital mortality and were removed, leaving 29 in the final model with random effect on the hospital level. Sixteen were associated with increased mortality risk, with the strongest associations coming from metastatic cancer (OR: 4.09, 95% CI: 3.98–4.21) and liver disease (OR: 3.83, 95% CI: 3.70–3.97). At the other end of the spectrum, 13 comorbidities were associated with a decreased risk of hospital mortality. The strongest of these were deficiency anaemia (OR: 0.54, 95% CI: 0.51–0.56) and obesity (OR: 0.59, 95% CI: 0.56–0.63). The adjusted coefficients were used to derive Swiss weights with a new maximum weight of 17, for metastatic cancer, and a new minimum of − 7, for deficiency anaemia (Table 2).

**Table 2:** Prevalence, adjusted odds ratio and weights from the (new) Swiss derivation sample and the van Walraven (VW) derivation sample [25]

Elixhauser comorbidities	Alive cohort (%)	Mortality cohort (%)	SMD	Adjusted odds ratio (95% CI)		Weights	
	Swiss derivation sample			VW^a^	Swiss	VW^a^	Swiss
^b^Derivation group	2,975,887 (97.7)	71,449 (2.3)					
Congestive heart failure	163,685 (5.5)	16,333 (22.9)	0.514	1.96 (1.85–2.07)	3.07 (3.00–3.14)	7	13
Cardiac arrhythmias	341,280 (11.5)	20,754 (29.0)	0.448	1.71 (1.62–1.80)	1.69 (1.66–1.73)	5	6
Valvular disease	117,450 (3.9)	6568 (9.2)	0.213	0.91 (0.82–0.99)	0.92 (0.89–0.95)	-1	-1
Pulmonary circulation disorders	53,292 (1.8)	4813 (6.7)	0.247	1.48 (1.34–1.62)	1.62 (1.57–1.68)	4	6
Peripheral vascular disorders	141,051 (4.7)	6912 (9.7)	0.192	1.26 (1.17–1.36)	1.27 (1.24–1.31)	2	3
Hypertension (uncomplicated)	676,609 (22.7)	15,692 (22.0)	0.019	–	0.69 (0.68–0.70)	0	−4
Hypertension (complicated)	218,656 (7.3)	11,003 (15.4)	0.256	–	0.79 (0.77–0.81)	0	−3
Paralysis	61,546 (2.1)	5153 (7.2)	0.246	1.93 (1.75–2.12)	2.60 (2.52–2.69)	7	11
Other neurological disorders	120,045 (4.0)	8011 (11.2)	0.273	1.83 (1.70–1.96)	2.45 (2.39–2.52)	6	10
Chronic pulmonary disease	170,770 (5.7)	8269 (11.6)	0.209	1.36 (1.29–1.44)	1.31 (1.27–1.34)	3	3
Diabetes, uncomplicated	245,817 (8.3)	9059 (12.7)	0.145	–	1.09 (1.06–1.11)	0	1
Diabetes, complicated	66,161 (2.2)	2763 (3.9)	0.096	–	0.89 (0.86–0.93)	0	−1
Hypothyroidism	126,062 (4.2)	3454 (4.8)	0.029	–	0.76 (0.74–0.79)	0	−3
Renal failure	289,047 (9.7)	20,526 (28.7)	0.497	1.63 (1.54–1.73)	2.06 (2.02–2.11)	5	8
Liver disease	49,916 (1.7)	5822 (8.1)	0.303	2.97 (2.73–3.22)	3.83 (3.7–3.97)	11	16
Peptic ulcer disease, excluding bleeding	5808 (0.2)	258 (0.4)	0.032	–	–	0	0
AIDS/HIV	2300 (0.1)	85 (0.1)	0.013	–	–	0	0
Lymphoma	25,049 (0.8)	1759 (2.5)	0.127	2.55 (2.31–2.81)	2.19 (2.07–2.31)	9	9
Metastatic cancer	119,667 (4.0)	18,907 (26.5)	0.657	3.30 (3.10–3.52)	4.09 (3.98–4.21)	12	17
Solid tumour without metastasis	268,298 (9.0)	24,046 (33.7)	0.631	1.47 (1.39–1.56)	2.36 (2.3–2.42)	4	10
Rheumatoid arthritis/collagen vascular diseases	47,305 (1.6)	1254 (1.8)	0.013	–	0.91 (0.86–0.97)	0	−1
Coagulopathy	90,551 (3.0)	9528 (13.3)	0.382	1.30 (1.22–1.40)	2.12 (2.07–2.18)	3	9
Obesity	68,155 (2.3)	1011 (1.4)	0.065	0.64 (0.53–0.77)	0.59 (0.56–0.63)	−4	−6
Weight loss	98,545 (3.3)	9527 (13.3)	0.369	1.85 (1.67–2.04)	1.67 (1.63–1.71)	6	6
Fluid and electrolyte disorders	257,618 (8.7)	17,440 (24.4)	0.434	1.61 (1.53–1.69)	1.58 (1.55–1.61)	5	5
Blood loss anaemia	19,759 (0.7)	685 (1.0)	0.033	0.81 (0.70–0.93)	0.66 (0.60–0.71)	−2	−5
Deficiency anaemia	72,290 (2.4)	1886 (2.6)	0.013	0.80 (0.71–0.90)	0.54 (0.51–0.56)	−2	−7
Alcohol abuse	96,708 (3.2)	3086 (4.3)	0.056	–	0.75 (0.72–0.78)	0	−3
Drug abuse	38,044 (1.3)	583 (0.8)	0.045	0.50 (0.42–0.60)	0.67 (0.61–0.73)	−7	−5
Psychoses	29,598 (1.0)	404 (0.6)	0.049	–	0.72 (0.65–0.79)	0	−4
Depression	173,898 (5.8)	3715 (5.2)	0.028	0.73 (0.67–0.80)	0.73 (0.70–0.75)	−3	−3

The total cohort percentages can exceed 100%, as each admission contributes to one or more comorbidities. Swiss weights are calculated by dividing the coefficient of each comorbidity by the coefficient in the model with the smallest absolute value (which is ‘diabetes uncomplicated’ with a coefficient of 0.084) and rounding to the nearest whole number

*Abbreviations: SMD* standardized mean difference between alive and mortality cohort, *VW*^*a*^ van Walraven, `–` excluded in the final model, ^b^Row percentage

### Validation and comparison of weighted comorbidity models

All three comorbidity weighting systems (Charlson, Elixhauser van Walraven and Swiss) indicated higher in-hospital mortality risk than the base model, showing the conditional interpretation of weights for each of the weighted models. Each model performed similarly across all years in validation groups as in the derivation groups. Overall, the c-statistic for the 6-year cohort were: 0.757 (95% CI: 0.755–0.759) for the base model, 0.850 (95% CI: 0.849–0.851) for Charlson, 0.863 (95% CI: 0.862–0.864) for VW Elixhauser and 0.867 (95% CI: 0.865–0.868) with Swiss Elixhauser. These c-statistics were similar in the development and validation cohorts. All differences and the rankings they established among models were statistically significant. (Additional file 1, Table S5). In comparison, the model with Swiss weights discrimination was slightly better with some c-statistic variability across the 6 years’ data.

**Fig. 1:**
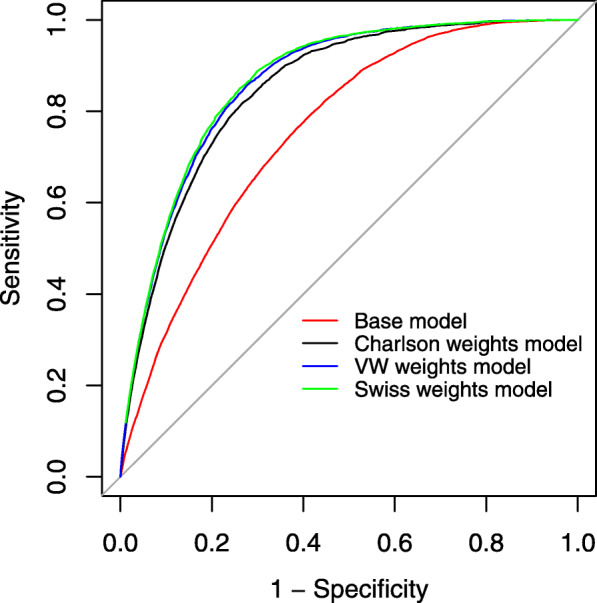
Receiver-operating characteristic (ROC) curves for generalized additive models predicting in-hospital mortality. Base model (AUC 0.757): age group, sex, hospital types; Charlson weights model (AUC 0.850): base and Charlson weights; VW weights model (AUC 0.863): base and Elixhauser/ van Walraven weights; Swiss weights model (AUC 0.867): base and Elixhauser/ Swiss weights. Straight diagonal line in the middle showing null model (AUC 0.500)

Additionally, 1% highest predicted value, showed the same order of the model’s performance from the observed mortality (base: 10.7%, Charlson: 18.5%, VW Elixhauser: 20.4%, Swiss Elixhauser: 20.9% (Table S6, Additional file 1). As shown in receiver-operating characteristic (ROC) curves (Fig. 1) the Swiss weights model’s discrimination was better than the Charlson’s or base model’s, and only slightly better than the van Walraven’s. The NRI confirm this picture (Table 3). Comparing the Swiss weights with VW weights showed an NRI of 1.6% (95%-CI: 1.3–2.0) with differences in predicted probabilities of mortality (among those who died) of 1.4% and differences in predicted probabilities of alive (among those who lived) by 0.02%.

**Table 3:** Comparison of Swiss weights model with Base, Charlson and VW weights models based on the Net Reclassification Improvement (NRI)

Derivation group					
Comparison models	NRI (95% CI)	Mortality increased Pr (Up|Case)	Alive increased Pr (Up|Ctrl)	Mortality decreased Pr (Down|Case)	Alive decreased Pr (Down|Ctrl)
Swiss weights vs. Base model	0.355 (0.352–0.357)	0.448 (0.445–0.450)	0.074 (0.074–0.074)	0.134 (0.133–0.136)	0.115 (0.115–0.116)
Swiss weights vs. Charlson weights model	0.049 (0.044–0.052)	0.297 (0.294–0.299)	0.058 (0.058–0.059)	0.251 (0.250–0.253)	0.062 (0.061–0.062)
Swiss weights vs. VW weights model	0.016 (0.013–0.020)	0.157 (0.155–0.159)	0.021 (0.021–0.022)	0.143 (0.140–0.145)	0.023 (0.023–0.024)

*Abbreviations: NRI* Net Reclassification Improvement with classification cut-off 0.023, *CI* confidence interval

*Pr (Up, Down) | (Case, Ctrl)* represents the proportion of patients whose predicted probabilities increased or decreased for in-hospital mortality and alive cohorts respectively

NRI = (Pr (Up|Case) - Pr (Down|Case)) + (Pr (Down|Ctrl) - Pr (Up|Ctrl))

Base model: age group, sex, hospital types

Charlson weights model: base and Charlson weights

VW weights model: base and Elixhauser/ van Walraven weights

Swiss weights model: base and Elixhauser/ Swiss weights

Finally, the sensitivity analysis using MDCs did not offer any improvements in the models’ performance.

## Discussion

This study used a six-year dataset of a multi-million-patient population to explore Charlson and Elixhauser comorbidities with different weightings to predict in-hospital mortality. We first derived a set of Swiss weightings for the 31 Elixhauser comorbidities using the national inpatient dataset. The analysis confirmed Charlson and Elixhauser comorbidities with van Walraven’s weights performance for mortality prediction, while the newly derived Swiss weightings slightly improved the mortality prediction for the 31 Elixhauser comorbidities.

Although, the optimized Swiss weightings performed only slightly better than the Charlson and Elixhauser-van Walraven sets they also supplied weights for eight Elixhauser comorbidities (e.g. diabetes, hypertension, and psychosis) eliminated by van Walraven et al. (2009) [25]. Of the risk-associated comorbidities retained in both the van Walraven and the Swiss weights, several comorbidities showed similar results, e.g., the highest odds ratios to metastatic cancer and liver disease. And regarding the comorbidities with negative associations, only small differences were observed between the van Walraven and Swiss weights (e.g., hypothyroidism or obesity were likely to be healthier).

From an epidemiological perspective, overall hospitalization mortality was only 2.3%, but in-hospital mortality is higher in patients with chronic diseases. Chronic diseases such as cancer, heart and liver diseases increase the risk of dying in hospitals, while certain other less severe diseases, (e.g., hypertension, anaemia and hypothyroidism) have a lower risk. This might be due to the relatively higher frequency of less severe diseases and some reported along with other acute conditions for the same patients. Furthermore, the interpretation of the algebraic sign of a single coefficient from such a joint model is mainly for the derivation of the weights, especially negative weights do not support the survival of the patients. These results are in line with those of Zellweger et al.’s [44] study using the Swiss national death registry of hospital inpatient data from 2010 to 2012. Furthermore, van Walraven et al.’s [25] study based on a single Canadian hospital’s records and Thompson et al., [21] using Maryland State inpatient data, showed similar results. These relations could insight the global burden of in-hospital mortality is due to rising chronic diseases.

The existing weighting systems [11, 13, 21, 25] represent data from a specific geographical region, patient group, or even limited numbers of hospitals or settings, matching the generalizability of these weighting systems remained difficult. As this study addresses such issues, with a large dataset representing the Swiss inpatient population, it provides Swiss comorbidity adjustments for the prediction of mortality or other health outcomes. The c statistics reported in our study (weighted models) are around 10% higher than those reported in van Walraven’s study [25]. Several reasons might explain this increase: the GAM modelling approach (with binomial family) including random effects contributing around 2% improvement in c statistic without random effect, the study cohort and hospital types included might raise the base model and largely the conditional interpretation of weightings effect raised c statistic of weighted models. With the new eight derivations, the additional eight significant variables might have played a role too. However, a slightly improved performance of the Swiss weights system suggests that it might be worthwhile to derive country- or region-specific comorbidity weights from representative patient populations.

C-statistics and ROCs are widely used to assess predictive performance. Nonetheless, one downside of comparing c-statistic and ROCs is that differences between c-statistics are often small, [45] as it was the case when we compared our new weights and van Walraven’s. Over the past decade, it has become common to use NRIs to compare different models’ performance, even though it might differ with the cut-offs taken for analysis [39, 46]. In our study, taking the same cut-offs for all models, NRI calculations confirmed the three weighting systems’ rankings i.e., Swiss, van Walraven and Charlson weights.

The primary strength of this study was the large sample size and the heterogeneity of the Swiss inpatient population across all general hospitals over 6 years, which made it representative of the entire country. To our knowledge, this study is the first to derive and validate Elixhauser weightings in Swiss hospital inpatient data. We used standard regression methodology for large datasets, including random effects at the hospital level, and internally validated our models. We also used accepted methods to modify our adjusted model into a Swiss weightings system that re-includes the association of several comorbidities (e.g., diabetes, hypertension, Psychoses) formerly excluded from the Elixhauser index in the VW study [35]. Despite differences in individual comorbidities’ prevalence and weightings, Charlson, Elixhauser/VW, and the Swiss weights performed well across the derivation, validation, and all-cases groups. We also used NRIs, allowing a robust comparison of model performance. Finally, the methods we applied were explicit and can be replicated by other researchers, who can adjust or control for patient comorbidity via their hospital and national databases. Moreover, the managerial utility could be done using this method by identifying high-risk patients for safe care and by evaluating hospitals performance based on the patient’s outcome.

Our study also has certain notable limitations. We derived our weights using statistical criteria, while clinical knowledge might be needed to determine each comorbidity’s value. Since we used codes assigned in routine data, the capture of the comorbidities could be influenced by other factors, such as physician and nurse documentation, code assignment accuracy, and the possibility that capture of comorbidities is biased towards those for which the Swiss DRG / MDC pays more [43, 47]. The negative coefficients/weights might be artefacts, as they are computed using routine data and coding of these is influenced by the main diagnose (e.g., deficiency anaemia, diabetes or hypertension are far more likely to be recorded when a patient had few other serious or acute problems). The direction of the coefficients is also driven by the joint adjusted model, which makes the interpretation of a single coefficient not meaningful. Moreover, some researchers believe current comorbidity indices are not suitable for use as predictors of patient-centred outcomes like rehabilitation, readmission, fee-for-services while weightings might differ in restricted cohorts, other outcomes and countries [27]. Additionally, Swiss data protection regulations prevented us from obtaining the inpatients’ exact age, we could not differentiate children exactly under 18 years and could not specify each year. This also might have influenced the predictive accuracy of the tested models.

## Conclusions

We found that Elixhauser/van Walraven weightings performed well in a large Swiss dataset and could derive Swiss weightings with statistically significant, yet with a small improvement in mortality prediction. Although the Swiss weightings showed slightly improved mortality predictions, we confirmed the validity of the Elixhauser/van Walraven weightings. The results provide evidence that Elixhauser/van Walraven weightings continue to be the preferred choice for weighting. In the Swiss context and possibly in countries with ICD-10 GM (German Modification) the derived weights are an option and to identify high-risk patients for safe care/treatment. Given access to similar data, researchers could use the methods described here to validate existing weightings such as van Walraven or derive their own country- or region-specific morbidity weights, although improvements might be small.

## Supplementary Information

**Additional file 1.**

## Data Availability

Upon application, the data that support the findings of this study are available from Federal Statistical Office (FSO), Switzerland.

## Abbreviations

ECI: Elixhauser Comorbidity Index
CCI: Charlson Comorbidity Index
ICD − 10 GM: International Classification of Diseases version-10 German Modification
DRGs: Diagnosis-Related Groups
SMD: Standardized Mean Differences
NRI: Net Reclassification Improvement
MDCs: Major Diagnostic Categories
ROC: Receiver-Operating Characteristic
FSO: Federal Statistical Office
SBK: Swiss Nurses’ Association
GAM: Generalized Additive Model
FMH: Swiss Medical Association
CI: Confidence Interval
