# Supplementary material for: Comparing Charlson and Elixhauser comorbidity indices with different weightings to predict in-hospital mortality: an analysis of national inpatient data
Source: BMC Health Serv Res. 2021 Jan 6;21:13. doi: 10.1186/s12913-020-05999-5 (PMC7786470; doi:10.1186/s12913-020-05999-5)
Supplement: Supplementary file 1 — Additional file 1. [file 12913_2020_5999_MOESM1_ESM.docx]

**Additional file 1. Supplementary figure and tables**

**Comparing Charlson, Elixhauser comorbidity indices with different weightings to predict in-hospital mortality: an analysis of national inpatient data**

**Fig. F1** Flowchart. National inpatient cases of all general hospitals for the analysis

**Table S1** The ICD-10 GM codes used in comorbidity package to classify the Charlson and the Elixhauser comorbidity indices

| **Charlson comorbidity index** | **ICD-10 codes** |
| --- | --- |
| Myocardial infarction | I21.x, I22.x, I25.2 |
| Congestive heart failure | I09.9, I11.0, I13.0, I13.2, I25.5, I42.0, I42.5 - I42.9, I43.x, I50.x, P29.0 |
| Peripheral vascular disease | I70.x, I71.x, I73.1, I73.8, I73.9, I77.1, I79.0, I79.2, K55.1, K55.8, K55.9, Z95.8, Z95.9 |
| Cerebrovascular disease | G45.x, G46.x, H34.0, I60.x - I69.x |
| Dementia | F00.x - F03.x, F05.1, G30.x, G31.1 |
| Chronic pulmonary disease | I27.8, I27.9, J40.x - J47.x, J60.x - J67.x, J68.4, J70.1, J70.3 |
| Rheumatic disease | M05.x, M06.x, M31.5, M32.x - M34.x, M35.1, M35.3, M36.0 |
| Peptic ulcer disease | K25.x - K28.x |
| Mild liver disease | B18.x, K70.0 - K70.3, K70.9, K71.3 - K71.5, K71.7, K73.x, K74.x, K76.0, K76.2 - K76.4, K76.8, K76.9, Z94.4 |
| Diabetes without chronic complication | E10.0, E10.1, E10.6, E10.8, E10.9, E11.0, E11.1, E11.6, E11.8, E11.9, E12.0, E12.1, E12.6, E12.8, E12.9, E13.0, E13.1, E13.6, E13.8, E13.9, E14.0, E14.1, E14.6, E14.8, E14.9 |
| Diabetes with chronic complication | E10.2 - E10.5, E10.7, E11.2 - E11.5, E11.7, E12.2 - E12.5, E12.7, E13.2 - E13.5, E13.7, E14.2 - E14.5, E14.7 |
| Hemiplegia or paraplegia | G04.1, G11.4, G80.1, G80.2, G81.x, G82.x, G83.0 - G83.4, G83.9 |
| Renal disease | I12.0, I13.1, N03.2 - N03.7, N05.2 - N05.7, N18.x, N19.x, N25.0, Z49.0 - Z49.2, Z94.0, Z99.2 |
| Any malignancy, including lymphoma and leukaemia, except malignant neoplasm of skin | C00.x - C26.x, C30.x - C34.x, C37.x - C41.x, C43.x, C45.x - C58.x, C60.x - C76.x, C81.x - C85.x, C88.x, C90.x - C97.x |
| Moderate or severe liver disease | I85.0, I85.9, I86.4, I98.2, K70.4, K71.1, K72.1, K72.9, K76.5, K76.6, K76.7 |
| Metastatic solid tumour | C77.x - C80.x |
| AIDS/HIV | B20.x - B22.x, B24.x |
|  |  |
| **Elixhauser comorbidity index** | **ICD-10 codes** |
| Congestive heart failure | I09.9, I11.0, I13.0, I13.2, I25.5, I42.0, I42.5 - I42.9, I43.x, I50.x, P29.0 |
| Cardiac arrhythmias | I44.1 - I44.3, I45.6, I45.9, I47.x - I49.x, R00.0, R00.1, R00.8, T82.1, Z45.0, Z95.0 |
| Valvular disease | A52.0, I05.x - I08.x, I09.1, I09.8, I34.x - I39.x, Q23.0 - Q23.3, Z95.2 - Z95.4 |
| Pulmonary circulation disorders | I26.x, I27.x, I28.0, I28.8, I28.9 |
| Peripheral vascular disorders | I70.x, I71.x, I73.1, I73.8, I73.9, I77.1, I79.0, I79.2, K55.1, K55.8, K55.9, Z95.8, Z95.9 |
| Hypertension (uncomplicated) | I10.x |
| Hypertension (complicated) | I11.x - I13.x, I15.x |
| Paralysis | G04.1, G11.4, G80.1, G80.2, G81.x, G82.x, G83.0 - G83.4, G83.9 |
| Other neurological disorders | G10.x - G13.x, G20.x - G22.x, G25.4, G25.5, G31.2, G31.8, G31.9, G32.x, G35.x - G37.x, G40.x, G41.x, G93.1, G93.4, R47.0, R56.x |
| Chronic pulmonary disease | I27.8, I27.9, J40.x - J47.x, J60.x - J67.x, J68.4, J70.1, J70.3 |
| Diabetes, uncomplicated | E10.0, E10.1, E10.9, E11.0, E11.1, E11.9, E12.0, E12.1, E12.9, E13.0, E13.1, E13.9, E14.0, E14.1, E14.9 |
| Diabetes, complicated | E10.2 - E10.8, E11.2 - E11.8, E12.2 - E12.8, E13.2 - E13.8, E14.2 - E14.8 |
| Hypothyroidism | E00.x - E03.x, E89.0 |
| Renal failure | I12.0, I13.1, N18.x, N19.x, N25.0, Z49.0 - Z49.2, Z94.0, Z99.2 |
| Liver disease | B18.x, I85.x, I86.4, I98.2, K70.x, K71.1, K71.3 - K71.5, K71.7, K72.x - K74.x, K76.0, K76.2 - K76.9, Z94.4 |
| Peptic ulcer disease, excluding bleeding | K25.7, K25.9, K26.7, K26.9, K27.7, K27.9, K28.7, K28.9 |
| AIDS/HIV | B20.x - B22.x, B24.x |
| Lymphoma | C81.x - C85.x, C88.x, C96.x, C90.0, C90.2 |
| Metastatic cancer | C77.x - C80.x |
| Solid tumour without metastasis | C00.x - C26.x, C30.x - C34.x, C37.x - C41.x, C43.x, C45.x - C58.x, C60.x - C76.x, C97.x |
| Rheumatoid arthritis/collagen vascular diseases | L94.0, L94.1, L94.3, M05.x, M06.x, M08.x, M12.0, M12.3, M30.x, M31.0 - M31.3, M32.x - M35.x, M45.x, M46.1, M46.8, M46. |
| Coagulopathy | D65 - D68.x, D69.1, D69.3 - D69.6 |
| Obesity | E66.x |
| Weight loss | E40.x - E46.x, R63.4, R64 |
| Fluid and electrolyte disorders | E22.2, E86.x, E87.x |
| Blood loss anaemia | D50.0 |
| Deficiency anaemia | D50.8, D50.9, D51.x - D53.x |
| Alcohol abuse | F10, E52, G62.1, I42.6, K29.2, K70.0, K70.3, K70.9, T51.x, Z50.2, Z71.4, Z72.1 |
| Drug abuse | F11.x - F16.x, F18.x, F19.x, Z71.5, Z72.2 |
| Psychoses | F20.x, F22.x - F25.x, F28.x, F29.x, F30.2, F31.2, F31.5 |
| Depression | F20.4, F31.3 - F31.5, F32.x, F33.x, F34.1, F41.2, F43.2 |

.x means all the sub-codes from the main ICD-10 code

**Table S2** Comorbidity index of Charlson, van Walraven and Swiss weights in the total study population

| Parameters | Alive cohort  (%) | Mortality cohort  (%) | SMD |
| --- | --- | --- | --- |
| Charlson index (Charlson weights) | | | 1.277 |
| 0 | 3,642,650 (61.2) | 17,465 (12.2) |  |
| 1–4 | 1,922,213 (32.3) | 72,876 (51.1) |  |
| >=5 | 387,142 (6.5) | 52,326 (36.7) |  |
| Elixhauser index (van Walraven weights) | | | 1.303 |
| <0 | 376,560 (6.3) | 1,216 (0.9) |  |
| 0 | 3,236,440 (54.4) | 14,311 (10.0) |  |
| 1–4 | 546,349 (9.2) | 9,300 (6.5) |  |
| >=5 | 1,792,656 (30.1) | 117,840 (82.6) |  |
| Elixhauser index (Swiss weights) | | | 1.322 |
| <0 | 1,154,573 (19.4) | 4,946 (3.5) |  |
| 0 | 2,561,720 (43.0) | 11,607 (8.1) |  |
| 1–4 | 426,880 (7.2) | 6,121 (4.3) |  |
| >=5 | 1,808,832 (30.4) | 119,993 (84.1) |  |

*Abbreviations: SMD*, standardized mean difference between alive and mortality cohort

| Charlson comorbidities | Total population | Swiss derivation sample | | |
| --- | --- | --- | --- | --- |
|  | N (%) | Alive cohort  (%) | Mortality cohort  (%) | SMD |
|  | 6,094,672 (100) | 2,975,887 (97.7) | 71,449 (2.3) |  |
| Myocardial infarction | 195,981 (3.2) | 91,930 (3.1) | 6,138 (8.6) | 0.236 |
| Congestive heart failure | 360,599 (5.9) | 163,685 (5.5) | 16,333 (22.9) | 0.514 |
| Peripheral vascular disease | 294,919 (4.8) | 141,051 (4.7) | 6,912 (9.7) | 0.192 |
| Cerebrovascular disease | 254,526 (4.2) | 118,841 (4.0) | 8,465 (11.8) | 0.294 |
| Dementia | 189,091 (3.1) | 89,536 (3.0) | 5,078 (7.1) | 0.188 |
| Chronic pulmonary disease | 358,407 (5.9) | 170,770 (5.7) | 8,269 (11.6) | 0.209 |
| Rheumatic disease | 83,179 (1.4) | 40,366 (1.4) | 1,061 (1.5) | 0.011 |
| Peptic ulcer disease | 34,831 (0.6) | 16,305 (0.5) | 1,144 (1.6) | 0.102 |
| Mild liver disease | 983,70 (1.6) | 46,028 (1.5) | 3,203 (4.5) | 0.172 |
| Diabetes without chronic complication | 523,029 (8.6) | 253,022 (8.5) | 9,333 (13.1) | 0.147 |
| Diabetes with chronic complication | 123,336 (2.0) | 59,298 (2.0) | 2,492 (3.5) | 0.092 |
| Hemiplegia or paraplegia | 133,663 (2.2) | 61,546 (2.1) | 5,153 (7.2) | 0.246 |
| Renal disease | 618,117 (10.1) | 289,180 (9.7) | 20,528 (28.7) | 0.497 |
| Any malignancy, including lymphoma and leukaemia, except malignant neoplasm of skin | 658,483 (10.8) | 302,801 (10.2) | 26,900 (37.6) | 0.680 |
| Moderate or severe liver disease | 29,538 (0.5) | 12,442 (0.4) | 2,331 (3.3) | 0.213 |
| Metastatic solid tumour | 276,426 (4.5) | 119,667 (4.0) | 18,907 (26.5) | 0.657 |
| AIDS/HIV | 4,894 (0.1) | 2,300 (0.1) | 85 (0.1) | 0.013 |

**Table S3** Prevalence of Charlson comorbidities from the total population and Swiss derivation sample

*Abbreviations: SMD*, standardized mean difference between alive and mortality cohort; The total cohort percentages can exceed 100%, as each admission contributes to one or more comorbidities.

**Table S4** Prevalence of Elixhauser comorbidities from the total and derivation sample

| Elixhauser comorbidities | Total population | Swiss derivation sample | | |
| --- | --- | --- | --- | --- |
|  | N (%) | Alive  cohort  (%) | Mortality cohort  (%) | SMD |
|  | 6,094,672 (100) | 2,975,887 (97.7) | 71,449 (2.3) |  |
| Congestive heart failure | 360599 (5.9) | 163,685 (5.5) | 16,333 (22.9) | 0.514 |
| Cardiac arrhythmias | 724927 (11.9) | 341,280 (11.5) | 20,754 (29.0) | 0.448 |
| Valvular disease | 248362 (4.1) | 117,450 (3.9) | 6,568 (9.2) | 0.213 |
| Pulmonary circulation disorders | 116734 (1.9) | 53,292 (1.8) | 4,813 (6.7) | 0.247 |
| Peripheral vascular disorders | 294919 (4.8) | 141,051 (4.7) | 6,912 (9.7) | 0.192 |
| Hypertension (uncomplicated) | 1383747 (22.7) | 676,609 (22.7) | 15,692 (22.0) | 0.019 |
| Hypertension (complicated) | 459645 (7.5) | 218,656 (7.3) | 11,003 (15.4) | 0.256 |
| Paralysis | 133663 (2.2) | 61,546 (2.1) | 5,153 (7.2) | 0.246 |
| Other neurological disorders | 256353 (4.2) | 120,045 (4.0) | 8,011 (11.2) | 0.273 |
| Chronic pulmonary disease | 358407 (5.9) | 170,770 (5.7) | 8,269 (11.6) | 0.209 |
| Diabetes, uncomplicated | 508108 (8.3) | 245,817 (8.3) | 9,059 (12.7) | 0.145 |
| Diabetes, complicated | 137602 (2.3) | 66,161 (2.2) | 2,763 (3.9) | 0.096 |
| Hypothyroidism | 258542 (4.2) | 126,062 (4.2) | 3,454 (4.8) | 0.029 |
| Renal failure | 617834 (10.1) | 289,047 (9.7) | 20,526 (28.7) | 0.497 |
| Liver disease | 111396 (1.8) | 49,916 (1.7) | 5,822 (8.1) | 0.303 |
| Peptic ulcer disease, excluding bleeding | 12012 (0.2) | 5,808 (0.2) | 258 (0.4) | 0.032 |
| AIDS/HIV | 4894 (0.1) | 2,300 (0.1) | 85 (0.1) | 0.013 |
| Lymphoma | 53679 (0.9) | 25,049 (0.8) | 1,759 (2.5) | 0.127 |
| Metastatic cancer | 276426 (4.5) | 119,667 (4.0) | 18,907 (26.5) | 0.657 |
| Solid tumour without metastasis | 583683 (9.6) | 268,298 (9.0) | 24,046 (33.7) | 0.631 |
| Rheumatoid arthritis/collagen vascular diseases | 97335 (1.6) | 47,305 (1.6) | 1,254 (1.8) | 0.013 |
| Coagulopathy | 200102 (3.3) | 90,551 (3.0) | 9,528 (13.3) | 0.382 |
| Obesity | 137896 (2.3) | 68,155 (2.3) | 1,011 (1.4) | 0.065 |
| Weight loss | 216541 (3.6) | 98,545 (3.3) | 9,527 (13.3) | 0.369 |
| Fluid and electrolyte disorders | 550094 (9.0) | 257,618 (8.7) | 17,440 (24.4) | 0.434 |
| Blood loss anaemia | 40893 (0.7) | 19,759 (0.7) | 685 (1.0) | 0.033 |
| Deficiency anaemia | 148305 (2.4) | 72,290 (2.4) | 1,886 (2.6) | 0.013 |
| Alcohol abuse | 199964 (3.3) | 96,708 (3.2) | 3,086 (4.3) | 0.056 |
| Drug abuse | 77640 (1.3) | 38,044 (1.3) | 583 (0.8) | 0.045 |
| Psychoses | 59909 (1.0) | 29,598 (1.0) | 404 (0.6) | 0.049 |
| Depression | 355765 (5.8) | 173,898 (5.8) | 3,715 (5.2) | 0.028 |

*Abbreviations: SMD*, standardized mean difference between alive and mortality cohort; The total cohort percentages can exceed 100%, as each admission contributes to one or more comorbidities.

|  | C Statistic (95% CI) | | | | | | | | |
| --- | --- | --- | --- | --- | --- | --- | --- | --- | --- |
|  | Derivation group | Validation groups | | | | | | Validation group | All cases |
|  | n = 3,047,336 | n_1_ = 491,962 | n_2_ = 496,684 | n_3_ = 504,741 | n_4_ = 514,267 | n_5_= 520,277 | n_6_ = 519,405 | n = 3,047,336 | N= 6,094,672 |
|  | All years  (2012–2017) | Year 1 | Year 2 | Year 3 | Year 4 | Year 5 | Year 6 | All years  (2012–2017) | All years  (2012–2017) |
| Base model | 0.757  (0.755–0.759) | 0.758  (0.752–0.759) | 0.758  (0.754–0.762) | 0.756  (0.753–0.760) | 0.752  (0.748–0.756) | 0.756  (0.752–0.759) | 0.750  (0.746–0.754) | 0.754  (0.753–0.754) | 0.757  (0.755–0.759) |
| Charlson weights model | 0.850  (0.847–0.851) | 0.849  (0.846–0.852) | 0.852  (0.849–0.855) | 0.854  (0.851–0.857) | 0.849  (0.846–0.852) | 0.854  (0.851–0.857) | 0.844  (0.841–0.847) | 0.849  (0.848–0.851) | 0.850  (0.849–0.851) |
| VW weights model | 0.863  (0.862–0.864) | 0.862  (0.859–0.865) | 0.866  (0.863–0.869) | 0.867  (0.864–0.869) | 0.863  (0.860–0.866) | 0.869  (0.866–0.872) | 0.862  (0.859–0.864) | 0.863  (0.862–0.864) | 0.863  (0.862–0.864) |
| Swiss weights model | 0.867  (0.865–0.868) | 0.865  (0.862–0.868) | 0.869  (0.866–0.871) | 0.871  (0.868–0.873) | 0.866  (0.863–0.869) | 0.872  (0.869–0.874) | 0.865  (0.862–0.867) | 0.866  (0.865-0.867) | 0.867  (0.865–0.868) |

**Table S5** Performance measures of the base, Charlson, van Walraven and Swiss weights models for in-hospital mortality in derivation, validation and all cases groups

*Abbreviations:* *VW* van Walraven, *CI* confidence interval

Base model: age groups, sex, hospital types

Charlson weights model: base and Charlson weights

VW weights model: base and Elixhauser/ van Walraven weights

Swiss weights model: base and Elixhauser/ Swiss weights

Note: Predicted probability of null model (c-statistic) is 0.50 of dying, which reflect the mean value of 2.3% from our data.

Table S6. The predicted and observed risk of patients from the highest percentage from the predicted values

| Highest percentage from the predicted values from the derivation sample | | | | |
| --- | --- | --- | --- | --- |
|  | 1% | 2% | 5% | 10% |
| Population-based weighting model (pre) | 0.332 | 0.260 | 0.176 | 0.124 |
| Population-based weighting model (obs) | 0.209 | 0.182 | 0.147 | 0.120 |
| VW weighting model (pre) | 0.319 | 0.251 | 0.172 | 0.123 |
| VW weighting model  (obs) | 0.204 | 0.181 | 0.137 | 0.119 |
| Charlson weighting model (pre) | 0.293 | 0.231 | 0.160 | 0.116 |
| Charlson weighting model (obs) | 0.185 | 0.167 | 0.137 | 0.112 |
| Base model (pre) | 0.110 | 0.099 | 0.085 | 0.072 |
| Base model (obs) | 0.107 | 0.094 | 0.084 | 0.072 |

Pre: mean predicted risk of dying, obs: mean observed risk of dying,

Note: Top 1% of admissions have higher risk of death.
